# Women’s preferences in the low-risk pregnancy management: Discrete Choice Experiments from Tuscany

**DOI:** 10.1093/eurpub/ckac131.435

**Published:** 2022-10-25

**Authors:** M Bonciani, I Corazza, A Ferrari, L Batinelli, F Pennucci

**Affiliations:** 1 Health and Management Laboratory, Sant'Anna School of Advanced Studies, Pisa, Italy; 2 School of Health Sciences, University of London, London, UK

## Abstract

During the last years, organizational models to assist women during pregnancy have changed. The physiological management of low-risk pregnancy is gaining momentum thanks to increased available evidence. Therefore, a need to assess women’s acceptance of these new proposals is emerging. This study aims to explore women’s preferences during pregnancy and childbirth. We enrolled women from the continuously active survey on the maternity pathway in Tuscany, Italy. We designed Two Discrete Choice Experiments (DCE) and administered them through web-based surveys. We sought to catch women’s preferences on health professionals to be involved, team-based or exclusive assistance, the physical proximity of healthcare services, and cost per service. We also explored women’s decision-making autonomy and pain management during labour and childbirth. Additionally, we investigated women’s information needs on the physiological model of low-risk pregnancy management through qualitative methods. Mixed logit models on the DCEs results show that women prefer being assisted during pregnancy and childbirth by healthcare services that are free of charge, easily accessible from a geographic point of view, and provided by a gynaecologist. The interaction effects reveal statistically significant differences for some sociodemographic variables of respondents and maternity pathway attributes. Besides, qualitative analyses highlight that women are interested in the physiological management of low-risk pregnancy according to their level of knowledge, confidence and safety feelings, and beliefs concerning non-medicalization and autonomy. The main findings of this study point out several implications for policy and managerial practice to effectively implement the physiological model of low-risk pregnancy management.

**Key messages:**

• This study proves that costs, travel distance, and type of health professional are the main determinants of women’s elicited preferences during pregnancy and childbirth.

• Policymakers and healthcare managers should consider these findings in organizing and providing maternal and childbirth health services to foster the physiological management of low-risk pregnancies.

